# Face mask type affects audiovisual speech intelligibility and subjective listening effort in young and older adults

**DOI:** 10.1186/s41235-021-00314-0

**Published:** 2021-07-18

**Authors:** Violet A. Brown, Kristin J. Van Engen, Jonathan E. Peelle

**Affiliations:** 1 Department of Psychological & Brain Sciences, Washington University in Saint Louis, St. Louis, USA; 2 Department of Otolaryngology, Washington University in Saint Louis, St. Louis, USA

**Keywords:** Face masks, Speech intelligibility, Subjective listening effort, Aging

## Abstract

Identifying speech requires that listeners make rapid use of fine-grained acoustic cues—a process that is facilitated by being able to see the talker’s face. Face masks present a challenge to this process because they can both alter acoustic information and conceal the talker’s mouth. Here, we investigated the degree to which different types of face masks and noise levels affect speech intelligibility and subjective listening effort for young (*N* = 180) and older (*N* = 180) adult listeners. We found that in quiet, mask type had little influence on speech intelligibility relative to speech produced without a mask for both young and older adults. However, with the addition of moderate (− 5 dB SNR) and high (− 9 dB SNR) levels of background noise, intelligibility dropped substantially for all types of face masks in both age groups. Across noise levels, transparent face masks and cloth face masks with filters impaired performance the most, and surgical face masks had the smallest influence on intelligibility. Participants also rated speech produced with a face mask as more effortful than unmasked speech, particularly in background noise. Although young and older adults were similarly affected by face masks and noise in terms of intelligibility and subjective listening effort, older adults showed poorer intelligibility overall and rated the speech as more effortful to process relative to young adults. This research will help individuals make more informed decisions about which types of masks to wear in various communicative settings.

**Supplementary Information:**

The online version contains supplementary material available at 10.1186/s41235-021-00314-0.

## Significance statement

Apart from numerous public health considerations, recent increases in face mask use have posed a challenge to our daily communication strategies, as face masks have the potential to disrupt both the auditory and visual speech produced by a talker. The current study quantifies the extent to which four different types of face masks affect speech intelligibility and subjective listening effort across various levels of background noise in young and older adults. Although the effects of face masks on speech communication have been increasingly scrutinized during the COVID-19 pandemic, this research has real-world implications beyond the pandemic, as face masks are also routinely used in many medical, industrial, and recreational settings. Our finding that surgical masks provide the least interference may be particularly informative for classroom instructors, clinicians, and other individuals who wear face masks in communicative settings. It is important to note, however, that intelligibility alone paints an incomplete picture of a listener’s experience. Indeed, a growing body of research indicates that factors that may not affect speech intelligibility (e.g., hearing aids, low levels of background noise) do affect listening effort—the cognitive resources necessary to comprehend speech. This research highlights this point, showing that some face masks are seen as particularly effortful even without background noise, where intelligibility does not differ from speech produced without a mask.

## Introduction

Understanding speech requires rapidly interpreting complex acoustic cues. Acoustic challenges like background noise make this process more difficult, and in these cases intelligibility is facilitated by being able to see the talker (Erber, 1972; Sumby & Pollack, 1954). Under many everyday listening conditions, successful communication therefore benefits from listeners’ access to both auditory and visual information.

Wearing a face mask interferes with the clarity of both signals: Materials used to decrease the transmission of pathogens and other airborne particles affect mouth movement and sound transmission, and face masks typically occlude the talker’s mouth, hiding important visual speech cues. Though the impact of face masks on speech understanding came to the forefront in 2020 during the coronavirus 19 (COVID-19) pandemic, face masks are also routinely used in many medical, industrial, and recreational settings. Despite their prevalence, relatively little is known about the extent to which various types of masks affect speech understanding.

Although several studies have measured the intelligibility of speech produced with a face mask, they have used either a single type of face mask (Cohn et al., 2021; Coyne et al., 1998; Mendel et al., 2008; Truong et al., 2021), a single signal-to-noise ratio (SNR; Bottalico et al., 2020; Cohn et al., 2021; Coyne et al., 1998; though see Toscano & Toscano, 2021), or presented speech only in quiet (Magee et al., 2020; Truong et al., 2021). Given the idiosyncrasies of how different types of face masks alter the acoustic speech signal and possible differences in how this interacts with the level of the background noise, our knowledge of how face masks affect speech understanding would benefit from research manipulating both mask type and noise level. It is especially important to include multiple noise levels given the robust evidence that visual information is differentially beneficial depending on noise level (Sumby & Pollack, 1954). In the current study, we therefore included five different face mask conditions (four face masks and one condition with no mask) and three levels of background noise.

In addition to assessing speech intelligibility across a range of noise levels and mask types, we also addressed whether aging affects the perception of speech produced with a face mask. Given the difficulties that older adults experience with speech identification in noise relative to young adults (e.g., Pichora-Fuller et al., 1995; Tun et al., 2002), we expected that older adults would show greater performance decrements than young adults when speech was produced with a face mask.

Although measuring speech intelligibility is a crucial step in understanding how listeners process speech produced with a face mask, intelligibility alone paints an incomplete picture of a listener’s experience. A growing body of work has demonstrated that even in conditions of equivalent intelligibility, differences in listening effort—the cognitive resources necessary to comprehend speech—may persist (Brown et al., 2020; Desjardins & Doherty, 2014; Koeritzer et al., 2018; Peelle, 2018; Rabbitt, 1968; Sarampalis et al., 2009; Ward et al., 2016). Thus, in addition to measuring speech intelligibility across a range of listening difficulties and mask types, we assessed subjective listening effort and subjective ratings of performance.

Finally, to begin exploring individual differences in how listeners are affected by speech produced with a face mask, we included a measure of depressive symptoms (Center for Epidemiologic Studies Depression Scale Revised (CESD-R); Eaton et al., 2004), which has been shown to be related to speech identification in some listening conditions (Chandrasekaran et al., 2015), and a measure of self-reported hearing ability (the 15-item Speech, Spatial, and Qualities of Hearing scale (15iSSQ); Moulin et al., 2019).

## Method

Stimuli, data, code for analyses, and the preregistered analysis plan are available at https://osf.io/atnv5/.

### Participants

We preregistered a sample size of 175 per age group, but increased this to 180 before collecting any data to enable a fully balanced design. To obtain our final sample of 180 young adults, we collected data from 222 participants. Forty-two participants were excluded based on preregistered exclusion criteria: Ten participants were excluded for having poor accuracy at the speech task (more than three standard deviations below the mean for any noise condition or any mask condition), eight reported having poor hearing for their age, 25 reported using speakers rather than headphones to complete the experiment, and one failed to complete the headphone questionnaire that followed the speech identification task. See Table 1 for demographic information.

**Table 1:** Demographic details for our final samples of 180 young adults and 180 older adults

Age (Years)	Gender
*Young adults*	
Median: 26 Mean: 26.5 SD: 5.1 Range: 18–35	Male: 52.8% Female: 45.6% Other: 0.6% No response: 1.1%
Older adults	
Median: 64 Mean: 64.6 SD: 4.1 Range: 59–77	Male: 30.0% Female: 70.0% Other: 0.0% No response: 0.0%

To obtain our final sample of 180 older adults, we also collected data from 222 participants. Eight were excluded for poor accuracy, 19 for having poor hearing for their age, 21 for using speakers rather than headphones, and one for reporting that they were younger than the minimum age allowed for participation, resulting in 42 participants who met exclusion criteria.

We used Gorilla Experiment Builder to create and host our experiment (Anwyl-Irvine et al., 2020), and participants were recruited and compensated via the online platform Prolific (www.prolific.co). The eligibility criteria listed in the experiment advertisement on Prolific indicated that participants must be native English speakers based in the United States with normal hearing and normal or corrected-to-normal vision, and they must use headphones. The experiment was completed only on desktop and laptop computers using Google Chrome. The experiment took approximately 45 min to complete, and participants were compensated at a rate of $10 per hour. The experimental protocol was approved by the Washington University in St. Louis Institutional Review Board.

### Sentence identification

**Fig. 1:**
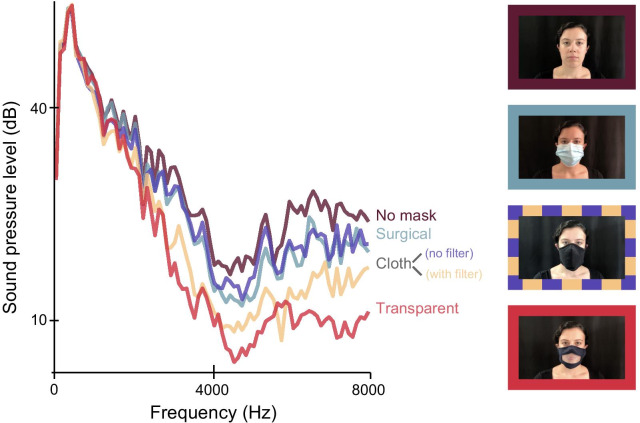
Average acoustic spectra across all sentences in each mask condition and example images of the talker in each condition. *Note.* The cloth mask looked the same with or without a filter

Speech stimuli consisted of 150 meaningful sentences (see Van Engen et al., 2012, 2014), each containing four key words (e.g., “The *gray mouse ate* the *cheese*”). Due to an error in programming, one of the sentences was presented twice to each participant. The second instance of this sentence was removed, resulting in 149 observations per participant. Stimuli were recorded by a female native speaker of American English in five mask conditions: no mask, a surgical mask, a black cloth mask with a paper filter (Safe Mate brand, 60% cotton, 40% polyester), the same cloth mask without a filter, and a cloth mask with a transparent plastic window (referred to as “transparent mask” from here forward). Stimuli were recorded across multiple days, but the same sentences were recorded in all mask conditions on a given day. The speaker, who has extensive experience recording stimuli for speech perception experiments, was instructed to maintain a consistent speaking style across conditions, and to avoid compensating for any reductions in audibility caused by face masks (e.g., by speaking more clearly or loudly in those conditions; Brumm & Zollinger, 2011; Lombard, 1911). The average spectra for each of the five mask conditions and images of the talker in each condition are shown in Fig. 1 (see Corey et al., 2020; Toscano & Toscano, 2021 for other demonstrations of acoustic attenuation produced by various types of face masks). Although it is possible that the speaker adjusted her speaking style differently for different face masks, all face masks attenuated the speech relative to the no mask condition, indicating that any compensation that occurred was not sufficient to overcome mask-induced attenuation.

The audio stimuli were recorded with a Blue Yeti microphone, and the visual stimuli were recorded with an iPhone 8 Plus (iOS 14.3) camera with 1920-by-1080 resolution at 30 frames per second. Audio stimuli were leveled by day such that the average amplitude of speech produced without a mask was set to the same level across days—that is, a consistent amplitude adjustment was applied to each mask condition on each day, allowing amplitude to vary by mask type. We opted to level in this way rather than match the amplitude of all stimuli because one of the ways masks may impair intelligibility is by reducing the amplitude of the speech (as demonstrated in Fig. 1), and we wanted to maintain this relevant between-mask difference. Prior to amplification, ambient noise was removed using Audacity (version 2.4.2). The audio and video files were then combined and the resulting files were spliced (using Adobe Premiere Pro CC 2017.1.1). We ensured that there was approximately 500 ms of silence before and after each sentence to allow the talker’s mouth to return to a closed position.

Next, each sentence was mixed with pink noise at two signal-to-noise ratios (SNRs), representing a moderate (− 5 dB SNR) and a hard (− 9 dB SNR) level of background noise. We opted to use pink noise as the background noise to avoid informational masking effects, and because it is more speech-like than white noise because the spectrum slopes downward (i.e., it does not selectively mask fricatives in the way white noise does). SNRs were created by holding the level of the speech constant and varying the level of the noise, using the average level of all speech files as the reference for adjusting the noise level. Thus, the actual SNR was slightly more difficult in conditions in which the face mask reduced overall speech amplitude—a decision that was made deliberately to mimic real-world listening. In addition to being presented in two different SNRs, sentences were also presented in quiet. When noise was present, it began 750 ms before the onset of the video and ended at the offset of the video. Video files were mixed with noise using ffmpeg software version 4.3.1 (ffmpeg.org).

Each of the 150 sentences was randomly assigned to one of 15 lists of 10 sentences, and sentence lists were counterbalanced across conditions. Thus, each sentence was presented in each of the 15 conditions (5 mask types * 3 noise levels) an equal number of times across participants, but each participant only heard each sentence in one condition. Each counterbalanced order was presented to 12 participants, resulting in a fully balanced design (15 list assignments * 12 participants = 180 total participants per age group). Condition was blocked, the order of the blocks was randomized across participants, and the order of the sentences within each block was also randomized.

### NASA task load index (NASA-TLX)

Subjective listening effort and performance were assessed via the NASA-TLX (Hart & Staveland, 1988), which contains six questions related to mental demand, physical demand, temporal demand, performance, effort, and frustration. Following each block of 10 sentences, participants were presented with the six questions, one at a time and in the same order as in the original paper (Hart & Staveland, 1988), and indicated their answer to the question with a response slider. Although number anchors were not visible to participants, the scale contains 21 values ranging from 1 to 21. Each participant provided a single response to each of the six items for each mask-by-noise condition, but we only analyzed the data for subjective effort (“How hard did you have to work to accomplish your level of performance?”) and performance (“How successful were you in accomplishing what you were asked to do?”). Following the scoring method of the original paper, higher numbers on the subjective effort question indicated greater perceived effort, and higher numbers on the subjective performance question indicated poorer perceived performance. Subjective performance questions were reverse coded prior to data analysis and visualization.

### Procedure

Before beginning the experiment, participants read a brief information sheet describing the task and completed a demographic questionnaire including questions about age, language background, sex, gender, race, ethnicity, and education. Next, participants completed the depression scale (CESD-R) and the assessment of self-reported hearing (15iSSQ; see Additional file 1 for details). They then completed the main sentence identification task followed by a brief headphone questionnaire about the type of output device they used to listen to the sentences (e.g., over-ear headphones, earbuds, external speakers). Prior to beginning the sentence task, participants completed five practice trials, one per mask type. Participants were asked to type their response in a text box that appeared after each sentence, and were encouraged to guess when unsure. After each block of 10 sentences (corresponding to one of the 15 conditions), participants responded to the six NASA-TLX questions for that block.

### Data analysis

Given the binary nature of the sentence intelligibility data and the fact that each sentence had four key words for scoring, data were analyzed using generalized linear mixed effects models assuming a (grouped) binomial outcome and a logit link function. All models with sentence intelligibility as an outcome included by-participant and by-sentence random intercepts, as well as by-participant and by-sentence random slopes for mask type and noise level. Deviations from this random effects structure for the subjective effort and performance analyses are explicitly noted. Responses were scored in R (see the accompanying R script for details) and were checked by an experimenter. Prior to scoring, extraneous punctuation was removed and all responses were converted to lowercase. Responses were scored as correct if they exactly matched the target word, were a homophone of the correct word, were a common misspelling or alternative spelling of the target word (e.g., “proffesor” for “professor” or “grey” for “gray”), or mismatched the target but the error was a single-letter addition, deletion, or substitution of the target (provided that mismatch was not also a word). Pluralizations and incorrect verb forms were not counted as correct. We maintained a copy of the raw responses in addition to the updated responses in the R script for transparency.

Data were processed via the *tidyverse* package version 1.3.0 (Wickham et al., 2019) and analyzed with Bayesian multilevel modeling via the *brms* package version 2.14.8 (Bürkner, 2017) using MCMC sampling and default priors in R version 4.0.3 (R Core Team, 2020). Each model reported below involved four chains of 6000 iterations each—2000 of which were warmup—resulting in 16,000 post-warmup posterior samples per model. All Rhat values were equal to 1.00, suggesting that no convergence issues were encountered during sampling. Models were compared by estimating the leave-one-out (LOO) cross-validation information criterion for each model using Pareto smoothed importance sampling—which provides an index of pointwise out-of-sample predictive accuracy for each model—and evaluating the difference in LOO between models and the standard error of the difference via the loo_compare() function from the *loo* package version 2.4.1 (Vehtari et al., 2020). Note that when reporting model comparisons, we report ΔELPD (expected log predictive density) rather than ΔLOO—which can be multiplied by − 2 to obtain ΔLOO if desired—to avoid unnecessarily reporting values on the deviance scale. These model comparisons were always conducted on models that differed in a single fixed effect but were otherwise identical; larger values of ΔELPD indicate larger differences in fit between the full and reduced models. We implemented a dummy coding scheme in which the quiet and no mask conditions were the reference levels.

## Results

### Speech intelligibility

**Fig. 2:**
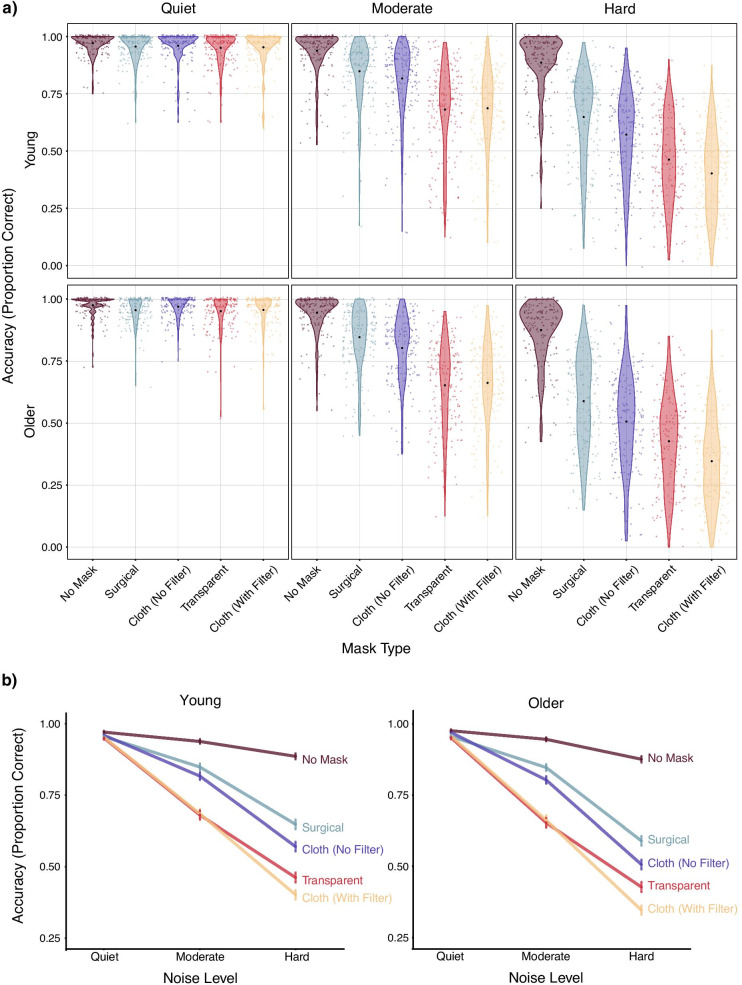
Sentence intelligibility in young and older adults. *Note.* **a** Young and older adults’ keyword intelligibility for each mask type by noise level. Black dots indicate the mean accuracy in each condition, and colored dots indicate means for individual participants. **b** Line graphs showing keyword intelligibility by noise level for each mask type in young and older adults. Error bars indicate ± two standard errors. Note that the bottom panel conveys the same information as the top panel but more clearly displays how noise affects intelligibility across mask types

Speech intelligibility data are shown in Fig. 2.

#### Age differences in speech intelligibility

For all analyses including age group as a fixed effect, we implemented a dummy coding scheme with young adults as the reference level. Model comparisons indicated that the three-way interaction between mask type, noise level, and age (*ΔELPD* = − 0.9, *ΔSE* = 5.4) and the two way interactions including age (mask type-by-age: *ΔELPD* = − 1.0, *ΔSE* = 1.8; noise level-by-age: *ΔELPD* = − 4.3, *ΔSE* = 2.3) provided negligible improvements in model fit. However, a model including age, mask type, and noise level (but no interactions) indicated that older adults had poorer speech intelligibility than young adults (B = − 0.24, CI = [− 0.40, − 0.08]); indeed, the proportion of posterior samples in which the estimate was negative (indicating poorer intelligibility for older adults) was greater than 0.998. Although we found no evidence for age differences in the extent to which mask type or noise level affected speech intelligibility, we report findings for young and older adults separately as stipulated in our preregistration.

#### Young adults

To assess whether mask type and noise level affect sentence intelligibility, we built a full model with fixed effects for mask type and noise level and compared this to two reduced models, each lacking one of the fixed effects of interest. Model comparisons indicated that both fixed effects substantially improved model fit (see Table 2). To assess pairwise comparisons of adjacent mask and noise conditions, we examined the summary output for the full model in conjunction with the hypothesis() function in the *brms* package, which can be used to conduct general linear hypothesis tests on any transformation of model parameters, obviating the need to relevel and refit the model multiple times. These indicated that intelligibility was worse in moderate noise relative to quiet (*B* = − 2.30, CI = [− 2.47, − 2.12]), and worse in hard relative to moderate noise (*B* = − 1.45, CI = [− 1.55, − 1.36]). Further, although intelligibility was best when the speaker was not wearing a face mask, the surgical mask led to better intelligibility than any other type of mask, followed by the cloth mask without a filter, the transparent mask, and finally the cloth mask with a filter. Zero was not contained in the 95% credible interval for any of these pairwise comparisons of adjacent conditions (e.g., surgical mask versus cloth mask without a filter; see R script for details).

**Table 2:** Change in LOO information criterion relative to the best fitting model without an interaction term for the sentence intelligibility analysis with young adults

	*ΔELPD*	*ΔSE*	Fixed and random effects
Mask and noise model	0.0	0.0	mask + noise + (1 + mask + noise|pid) + (1 + mask + noise|sentence)
Mask model	− 31.2	8.3	mask + (1 + mask + noise|pid) + (1 + mask + noise|sentence)
Noise model	− 28.4	7.7	noise + (1 + mask + noise|pid) + (1 + mask + noise|sentence)

*Note.* pid = participant identification number

Next, we compared a model including the interaction between mask type and noise level to a model lacking the interaction; the interaction term also substantially improved model fit relative to the standard error of the difference in fits (*ΔELPD* = − 237.5; *ΔSE* = 30.4). Thus, the effect of mask type on speech intelligibility depends on noise level (Fig. 2a), or, equivalently, the effect of noise level depends on mask type (Fig. 2b). Overall, the interaction indicates that face masks impair intelligibility to a greater extent as the level of the background noise increases. Indeed, the only mask that differed from the no mask condition in quiet was the transparent mask (*B* = − 0.46, CI = [− 0.76, − 0.15]).

#### Older adults

The analyses for older adults mirrored those reported above. Both mask type and noise level improved model fit (Table 3), and the pattern of results was the same as that in young adults: Intelligibility declined as noise level increased, and face masks impaired performance to varying degrees, with surgical masks providing the least interference and cloth masks with filters providing the most interference (Fig. 2). Performance was worse in moderate noise than in quiet (*B* = − 2.52, CI = [− 2.72, − 2.33]) and worse in hard noise than in moderate noise (*B* = − 1.62, CI = [− 1.72, − 1.51]), and zero was not contained in the 95% credible interval for any comparison of adjacent mask conditions. Further, model comparisons provided evidence for an interaction effect (*ΔELPD* = − 262.9, *ΔSE* = 31.2), indicating that the detrimental effects of face masks on speech intelligibility were more pronounced in higher levels of noise (Fig. 2). Just as with the young adults, the only mask that differed from the no mask condition in quiet was the transparent mask (*B* = − 0.46, CI = [− 0.82, − 0.10]).

**Table 3:** Change in LOO information criterion relative to the best fitting model without an interaction term for the sentence intelligibility analysis with older adults

	*ΔELPD*	*ΔSE*	Fixed and random effects
Mask and noise model	0.0	0.0	mask + noise + (1 + mask + noise|pid) + (1 + mask + noise|sentence)
Mask model	− 23.8	7.3	mask + (1 + mask + noise|pid) + (1 + mask + noise|sentence)
Noise model	− 29.9	8.6	noise + (1 + mask + noise|pid) + (1 + mask + noise|sentence)

*pid* = participant identification number

### Subjective listening effort (NASA-TLX)

**Fig. 3:**
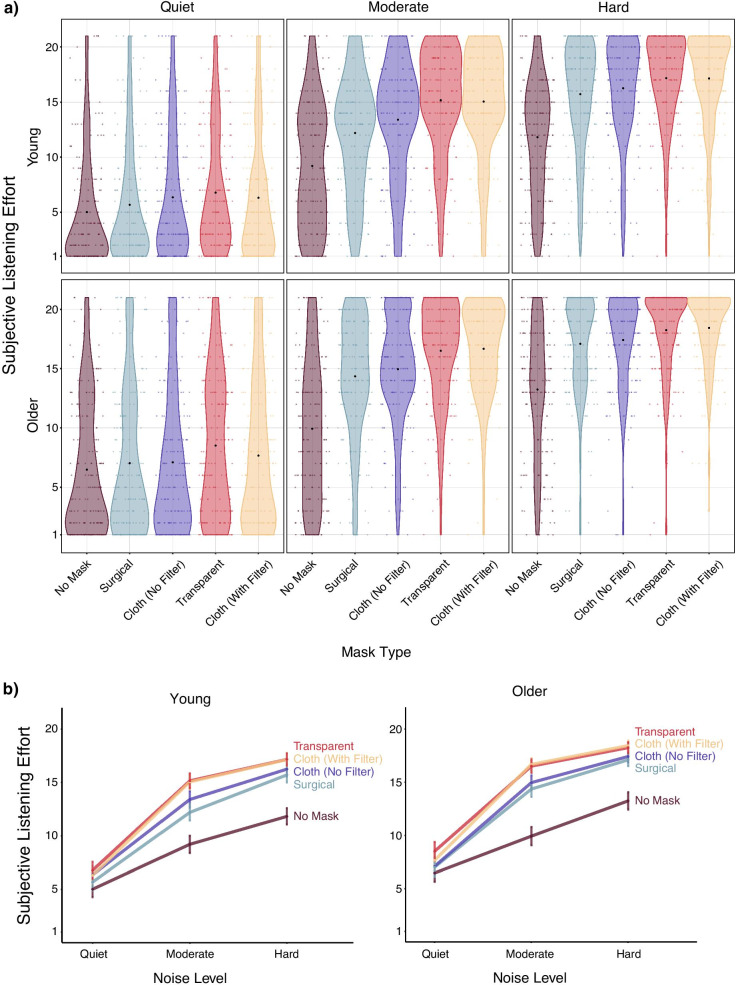
Subjective listening effort in young and older adults. Note. **a** Young and older adults’ subjective listening effort ratings for each mask type by noise level. Black dots indicate the mean effort rating in each condition, and colored dots indicate means for individual participants. **b** Line graphs showing subjective listening effort ratings by noise level for each mask type in young and older adults. Error bars indicate ± two standard errors. Note that the bottom panel conveys the same information as the top panel but more clearly displays how noise affects subjective effort across mask types. Responses range from 1–21

Subjective listening effort data are shown in Fig. 3. Unless otherwise noted, analysis of the subjective listening effort data mirrored the analyses for the sentence identification data. NASA-TLX data were analyzed assuming a Gaussian distribution with an identity link function, and the random effects structure included by-participant random intercepts and slopes for noise level (but not mask type; see https://osf.io/g2j94 for details). We did not include item random effects in the NASA-TLX analyses because participants were told to respond to the previous set of sentences rather than to individual items. Given that the pattern of results for the subjective performance analyses were the same as those for the subjective effort analyses for both age groups, those data are reported in Additional file 1.

#### Age differences in subjective effort

Model comparisons indicated that the three-way interaction between mask type, noise level, and age (*ΔELPD* = − 2.0, *ΔSE* = 4.0) and both two-way interactions between age and either mask type (*ΔELPD* = − 1.9, *ΔSE* = 2.2) or noise level (*ΔELPD* = − 0.3, *ΔSE* = 1.0) provided negligible improvements in model fit. However, a model that included mask type, noise level, and age indicated that older adults rated the speech as subjectively more effortful to process than young adults (*B* = 1.26, CI = [0.57, 1.95]). Indeed, the proportion of posterior samples in which the estimate for subjective effort was positive (indicating greater perceived effort for older adults) was greater than 0.999. Although we found no evidence for age differences in the extent to which mask type or noise level affected subjective effort ratings, we report findings for young and older adults separately below, as stipulated in our preregistration.

#### Young adults

Model comparisons provided evidence for effects of both mask type and noise level (Table 4). Subjective effort ratings showed a similar pattern of results to the intelligibility data: Effort ratings were higher in the moderate noise level relative to quiet (*B* = 6.98, CI = [6.39, 7.58]) and in the hard relative to the moderate noise level (*B* = 2.61, CI = [2.26, 2.95]). Further, effort ratings were lowest when the talker was not wearing a mask, ratings were higher for the surgical mask, and higher still for the cloth mask without a filter (zero was not contained in the 95% credible interval for any of these pairwise comparisons). Effort ratings were highest when the talker wore either a transparent mask or a cloth mask with a filter, which did not differ from one another (*B* = − 0.19, CI = [− 0.58, 0.19]).

**Table 4:** Change in LOO information criterion relative to the best fitting model without an interaction term for the subjective listening effort analysis in young adults

	*ΔELPD*	*ΔSE*	Fixed and random effects
Mask and noise model	0.0	0.0	mask + noise + (1 + noise|pid)
Mask model	− 18.1	6.7	mask + (1 + noise|pid)
Noise model	− 311.1	26.1	noise + (1 + noise|pid)

*pid* = participant identification number

Additional model comparisons indicated that the interaction between mask type and noise level provided a substantial improvement in model fit (*ΔELPD* = − 77.1; *ΔSE* = 13.4). As in the intelligibility analysis, the interaction indicated that the detrimental effects of face masks on subjective effort were exacerbated in more difficult listening conditions (Fig. 3). However, it is worth noting that although intelligibility was unaffected by face masks in quiet (with the exception of the transparent mask), participants rated all four masked conditions as subjectively more effortful than the no mask condition in quiet (none of the 95% credible intervals contained zero).

#### Older adults

Analyses of the older adult data mirrored those described for the young adult data. Model comparisons indicated that both mask type and noise level substantially improved model fit (Table 5). The subjective data again showed a similar pattern to the intelligibility data: Participants reported expending greater effort in the moderate noise level relative to quiet (*B* = 7.14, CI = [6.55, 7.73]) and in the hard relative to the moderate noise level (*B* = 2.41, CI = [2.05, 2.76]). The 95% credible interval did not contain zero for the pairwise comparisons of adjacent mask conditions (see R script for details) with the exception of the cloth mask without a filter and the surgical mask (*B* = 0.33, CI = [− 0.06, 0.72]), as well as the cloth mask with a filter and the transparent mask (*B* = − 0.16, CI = [− 0.57, 0.24]). Finally, model comparisons provided evidence for an interaction effect (*ΔELPD* = − 87.3, *ΔSE* = 16.1) such that effort ratings were more affected by face masks in higher levels of background noise (Fig. 3). Further, in the quiet condition, older adults rated the transparent mask (*B* = 2.03, CI = [1.28, 2.78]) and cloth mask with a filter (*B* = 1.19, CI = [0.44, 1.93]) as subjectively more effortful than the no mask condition, but ratings did not differ from the no mask condition for either the surgical mask (*B* = 0.55, CI = [− 0.21, 1.29]) or the cloth mask without a filter (*B* = 0.62, CI = [− 0.13, 1.37]; note that this differs from the pattern of results observed in young adults).

**Table 5:** Change in LOO information criterion relative to the best fitting model without an interaction term for the subjective listening effort analysis in older adults

	*ΔELPD*	*ΔSE*	Fixed and random effects
Mask and noise model	0.0	0.0	mask + noise + (1 + mask|pid)
Mask model	− 15.0	5.4	mask + (1 + mask|pid)
Noise model	− 302.9	26.5	noise + (1 + mask|pid)

*pid* = participant identification number

### Depression inventory (CESD-R) and self-reported hearing ability (15iSSQ)

We did not find any evidence that depression levels or self-reported hearing ability were related to the extent to which face masks or noise level affected sentence identification in young or older adults. Details are provided in Additional file 1.

## Discussion

In the current experiment, we investigated the degree to which various types of face masks and noise levels affect audiovisual speech understanding. We found that although face masks had little effect on speech intelligibility in quiet, intelligibility dropped substantially with even moderate levels of background noise. Indeed, for the transparent mask and the cloth mask with a filter, accuracy dropped approximately 30% in − 5 dB SNR noise relative to quiet. We also showed that although different types of face masks provide varying levels of interference, every type of face mask impairs speech intelligibility when background noise is present. These findings were broadly as expected, and consistent with other reports in the literature (Corey et al., 2020; Mendel et al., 2008; Toscano & Toscano, 2021).

In addition to speech intelligibility, we also assessed subjective effort during listening using the NASA-TLX. It is important to emphasize that subjective effort is only one measure of listening effort, and that various listening effort measures often do not agree with one other (Strand et al., 2018). However, subjective effort has been shown to affect decision making across domains (Crawford et al., 2021; McLaughlin et al., 2021; Westbrook & Braver, 2015), making it an appealing way to assess listener experience. We found that subjective effort ratings largely paralleled the intelligibility data—effort increased with noise and differentiated between mask types. Of particular note is that even in quiet, where intelligibility was extremely high for all mask types and only the transparent mask led to reduced intelligibility in both age groups, subjective effort was greater in most of the masked conditions relative to unmasked speech.

Consistent with previous research, we found that older adults had lower intelligibility overall and reported greater listening effort compared to young adults. Surprisingly, we did not find any evidence for notable age differences in how the type of face mask or background noise level affected audiovisual speech processing. Furthermore, we did not see any effect of self-reported hearing ability on speech intelligibility. One potential reason for these findings is that the population of older adults actively seeking online studies may systematically differ from the population typically recruited in laboratory settings. Another important point is that participants controlled presentation levels; listeners with poorer hearing may have increased the volume on their output devices more than those with better hearing, which may have reduced the effects of noise on age-related differences in speech identification. In any case, the degree to which older adults may be differentially affected by face masks during communication requires additional study.

Although we did not find evidence that either depression level or self-reported hearing ability was related to speech intelligibility (see Additional file 1), the violin plots nonetheless show substantial inter-individual variability in both sentence identification (Fig. 2) and subjective listening effort (Fig. 3). Thus, the extent to which individuals are affected by various types of face masks is a fruitful avenue for future research.

Our experiment is conceptually similar to a recent experiment by Toscano and Toscano (2021), which found modest decreases in speech intelligibility that differed by mask type. However, it is worth emphasizing several key differences between the two studies. First and foremost, Toscano and Toscano (2021) studied auditory-only speech, which allowed them to focus on acoustic differences across conditions at the expense of audiovisual cues typically present in everyday conversation. Our experiment also builds on previous work by assessing the effects of face masks on speech intelligibility in difficult listening conditions. Given that audiovisual benefit differs across noise levels (e.g., Erber, 1969), face masks may also exert different influences on speech intelligibility at different noise levels, so it is necessary to assess these effects across a wide range of listening conditions. Finally, in the current study we also included a subjective measure of listening effort to complement intelligibility scores, and assessed whether the effects of face masks differed between young and older adults. Our results therefore build on previous research by extending the basic research question—*how do face masks affect speech perception?*—to a variety of listening conditions, outcomes, and age groups.

Our findings highlight several additional considerations regarding face masks and communication. We found that the transparent face mask and the cloth mask with a filter led to the poorest intelligibility and highest effort ratings for both age groups, consistent with the fact that these two types of face masks provided the most attenuation, particularly at high frequencies (Fig. 1). Thus, a straightforward conclusion would be that avoiding these face masks would, on average, improve communication effectiveness, and that among the masks we tested, a surgical mask would generally lead to the best speech understanding.

The finding that the transparent mask led to poor intelligibility and high listening effort may at first seem counterintuitive because the listener can see the talker’s mouth (e.g., Erber, 1969; Sumby & Pollack, 1954). However, in our experience, condensation quickly formed on the transparent mask, obscuring visual information. Further, the acoustic challenge created by the impermeable plastic appears to largely outweigh the benefit of seeing the talker’s mouth for listeners with normal hearing. It is important to emphasize that for listeners who do *not* have normal hearing, the ability to see a talker’s mouth may be essential (Chodosh et al., 2020; McKee et al., 2020; Saunders et al., 2021). Indeed, given that the transparent mask provided even more attenuation than the cloth mask with a filter, yet intelligibility and effort were comparable in these two conditions, it may be that listeners benefitted enough from the availability of visual cues to make up for the additional acoustic attenuation and bring performance to the level of that for speech produced with a filtered cloth mask. This benefit may be especially important for individuals with hearing difficulty. Thus, our results should not be taken to suggest that one type of mask will be the best in all situations; instead, specific populations may differentially benefit from various mask types. Regardless of the type, our results indicate that when wearing a face mask is necessary, it is especially important to attempt to minimize noise in the environment to facilitate efficient speech communication.

## Supplementary Information

**Additional file 1**. Analyses of the CESD-R, 15iSSQ, and subjective performance data, as well as additional tables.

## Data Availability

All data, materials, and code for analyses are available via the OSF at https://osf.io/atnv5/.
