# Supplementary material for: Face mask type affects audiovisual speech intelligibility and subjective listening effort in young and older adults
Source: Cogn Res Princ Implic. 2021 Jul 18;6:49. doi: 10.1186/s41235-021-00314-0 (PMC8286438; doi:10.1186/s41235-021-00314-0)
Supplement: Supplementary file 1 — Additional file 1. Analyses of the CESD-R, 15iSSQ, and subjective performance data, as well as additional tables. [file 41235_2021_314_MOESM1_ESM.pdf]

This is the Supplementary Materials document accompanying the manuscript: Face Mask Type Affects Audiovisual Speech Intelligibility and Subjective Listening Effort in Young and Older Adults (Violet Brown, Kristin Van Engen, & Jonathan Peelle).

Individual Differences Analyses

To begin exploring individual differences in how face masks affect speech intelligibility, we included two auxiliary measures in this experiment. We included a measure of depressive symptomology because previous work has shown poorer speech identification in one-talker babble for individuals with elevated depressive symptoms relative to those with low depressive symptoms (Chandrasekaran et al., 2015). We therefore assessed whether depression scores were related to speech identification performance in young and older adults, and whether individuals reporting more depressive symptoms showed larger performance decrements when the speech was produced with a face mask relative to those with lower depression levels.

We also included a measure of self-reported hearing ability because this experiment was conducted online and audiological examinations were therefore unavailable, and previous work has shown that hearing ability may relate to cognitive demands during listening (Koeritzer et al., 2018; Lee et al., 2018; McCoy et al., 2005; Tun et al., 2009). We expected that self-reported hearing ability would be associated with differences in both speech intelligibility.

Method

Center for Epidemiologic Studies Depression Scale Revised (CESD-R)

To assess depressive symptoms, we implemented a revised version of the CESD (Radloff, 1977). The CESD-R (Eaton et al., 2004) contains 20 questions, but we removed two questions relating self-harm and suicide, resulting in an 18-item scale. Participants were presented with 18 statements (e.g., “My appetite was poor”) and responded by selecting one of six options: Not at all or less than 1 day, 1–2 days, 3–4 days, 5–7 days, nearly every day for two weeks, prefer not to answer. Scores ranged from 0 to 3 for each question (0 = Not at all or less than 1 day, 1 = 1–2 days, 2 = 3–4 days, 3 = 5–7 days or nearly every day for two weeks). Scores were calculated by summing the responses across all questions for each participant. Higher scores indicate higher depression levels.

15-Item Speech, Spatial, and Qualities of Hearing (15iSSQ) Scale

To assess subjective hearing ability, we used a short-form version of the Speech, Spatial, and Qualities of Hearing (SSQ) scale, which is intended to measure subjective hearing ability in a variety of domains (Gatehouse & Noble, 2004). The reduced version—the 15iSSQ (Moulin et al., 2019)—contains 15 items divided into three domains: five relating to speech hearing (e.g., “You are in conversation with one person in a room where there are many other people talking. Can you follow what the person you are talking to is saying?”), five relating to spatial hearing (e.g., “You are outside. A dog barks loudly. Can you tell immediately where it is, without having to look?”), and five relating to other qualities of hearing (e.g., “When you listen to music, does it sound clear and natural?”). Participants rated their agreement with the statement on a scale from 0 (not at all) to 10 (perfectly) using a response slider, and the overall score was calculated by averaging responses to the 15 items for each participant. We additionally calculated a speech subscore by averaging responses to the five speech items for each participant. Higher scores indicate better perceived hearing ability.

Results

Depression Inventory (CESD-R)

To assess whether depressive symptomology was related to speech intelligibility, we compared a model including mask type, noise level, and CESD-R score to a model lacking CESD-R score, separately for young and older adults. For each age group, we additionally assessed whether CESD-R interacted with mask type and noise level by comparing a model including interactions between CESD-R scores and mask and noise to reduced models lacking one of the interactions. This allowed us to assess whether the observed effects of face masks and noise level on speech intelligibility reported above were moderated by depressive symptomology. The CESD-R effect was tested by comparing a model with mask type, noise level, and CESD-R score (but no interactions) to a reduced model lacking a fixed effect for CESD-R. Interactions were tested by comparing a model with all two-way interactions to a model lacking the particular interaction of interest.

Age Differences in CESD-R Scores. Given that CESD-R scores are calculated by summing responses to obtain a single total score for each participant, we used fixed-effects only Bayesian regression to assess whether depression scores differed by age group. We used a dummy coding scheme with young adults as the reference level. Results showed that older adults had lower levels of depression than young adults (B = -4.97, CI = [-7.22, -2.75]; Table S8).

Young Adults. Model comparisons indicated that the improvement in model fit by including CESD-R score was negligible relative to the standard error of the difference in fit (ΔELPD = -0.1, ΔSE = 0.9). Additional model comparisons indicated that the improvements in fit by including the interaction between CESD-R and mask type (ΔELPD = -1.4, ΔSE = 2.9) or the interaction between CESD-R and noise level (ΔELPD = -0.1, ΔSE = 1.9) were negligible.

Older Adults. As with young adults, we did not find any evidence for effects of CESD-R (ΔELPD = -1.3, ΔSE = 1.2), the interaction between CESD-R and mask type (ΔELPD = -1.6, ΔSE = 2.2), or the interaction between CESD-R and noise level (ΔELPD = -2.3, ΔSE = 1.2) on speech intelligibility.

Self-Reported Hearing Ability (15iSSQ)

The analyses including the 15iSSQ data mirrored those described above for the CESD-R data: we examined the effect of 15iSSQ score, as well as the interaction between 15iSSQ score and both mask type and noise level separately for young and older adults. These analyses were also conducted on 15iSSQ speech subscores.

Age Differences in 15iSSQ scores. Given that 15iSSQ scores are calculated by averaging responses to obtain a single total score and a single speech subscore for each participant, we assessed whether young and older adults differed in self-reported hearing ability via fixed-effects only Bayesian regression. We again implemented a dummy coding scheme with young adults as the reference level. Surprisingly, older adults had better self-reported hearing ability than young adults as measured by total 15iSSQ score (B = 0.40, CI = [0.14, 0.66]; Table S9). The two groups did not differ on the speech subscore (B = 0.18, CI = [-0.14, 0.50]).

Young Adults. Models including either the total score (Δ*ELPD* = -1.1, Δ*SE* = 1.0) or the speech subscore (Δ*ELPD* = -1.3, Δ*SE* = 1.0) provided negligible improvements in fit relative to a reduced model lacking 15iSSQ scores. Further, neither the total score nor the speech subscore interacted with mask type

(total: Δ*ELPD* = -2.6, Δ*SE* = 1.3; speech: Δ*ELPD* = -2.1, Δ*SE* = 1.5)[1] or noise level (total: Δ*ELPD* = -0.3, Δ*SE* = 1.1; speech: Δ*ELPD* = -0.3, Δ*SE* = 1.0).

Older Adults. We did not find any evidence that the total score (Δ*ELPD* = -1.1, Δ*SE* = 1.6) or the speech subscore (Δ*ELPD* = -3.8, Δ*SE* = 2.2) affected speech intelligibility in older adults. Neither score interacted with mask type (total: Δ*ELPD* = -0.9, Δ*SE* = 2.5; speech: Δ*ELPD* = -2.3, Δ*SE* = 3.1) or noise level (total: Δ*ELPD* = -0.2, Δ*SE* = 1.1; speech: Δ*ELPD* = -1.3, Δ*SE* = 1.3).

Discussion

We found no evidence that depression or self-reported hearing were related to speech intelligibility, nor did they interact with either mask type or noise level in either age group. Previous research has demonstrated a relationship between speech intelligibility and depression levels, but that study only found evidence for this effect in one-talker babble (Chandrasekaran et al., 2015); it is therefore not surprising that the relationship did not emerge in the steady-state pink noise we used here. One might expect, however, that individuals with poorer self-reported hearing ability would be more affected by face masks and background noise than those with good self-reported hearing, but we did not find evidence for either relationship. The questions on the 15iSSQ tap into high-level hearing abilities—such as speech stream segregation and auditory localization—as opposed to hearing thresholds, so future research could assess whether pure tone thresholds moderate the effects reported here.

Subjective Performance Analyses

Although higher scores on the NASA-TLX subjective performance question indicate poorer perceived performance, responses have been reverse coded for all analyses and figures for ease of interpretation (i.e., higher scores indicate better perceived performance). Subjective performance data are shown in **Figure S1**.

Age Differences in Subjective Performance

The three-way interaction between mask type, noise level, and age (Δ*ELPD* = -3.8, Δ*SE* = 3.4) and both two-way interactions between age and either mask type (Δ*ELPD* = -5.3, Δ*SE* = 4.4) or noise level (Δ*ELPD* = -0.7, Δ*SE* = 1.5) provided negligible improvements in model fit. However, an effect of age emerged in a model that also included mask type and noise level such that older adults perceived that they performed more poorly than young adults (B = -0.82, CI = [-1.30, -0.35]). Indeed, the proportion of posterior samples in which the estimate was negative (indicating poorer perceived performance for older adults) was greater than .999. Although we found no evidence for age differences in the extent to which mask type or noise level affected subjective performance ratings, we report findings for young and older adults separately below, as stipulated in our preregistration.

Figure S1. Subjective performance. **a)** *Young and older adults' by-participant subjective performance ratings for each mask type by noise level. Black dots indicate the mean performance rating in each condition and colored dots indicate means for individual participants.* **b)** *Line graph showing subjective performance ratings by noise level for each type of mask in young and older adults. Error bars indicate ± two standard errors. Note that the bottom panel conveys the same information as the top panel but more clearly displays how noise affects subjective performance across masks. Responses ranged from 1–21.*

[1] Note that model fit indices provided weak evidence for an interaction between mask type and 15iSSQ total score, but the 95% credible interval for coefficient estimates for every mask included zero. See accompanying R script.

a)

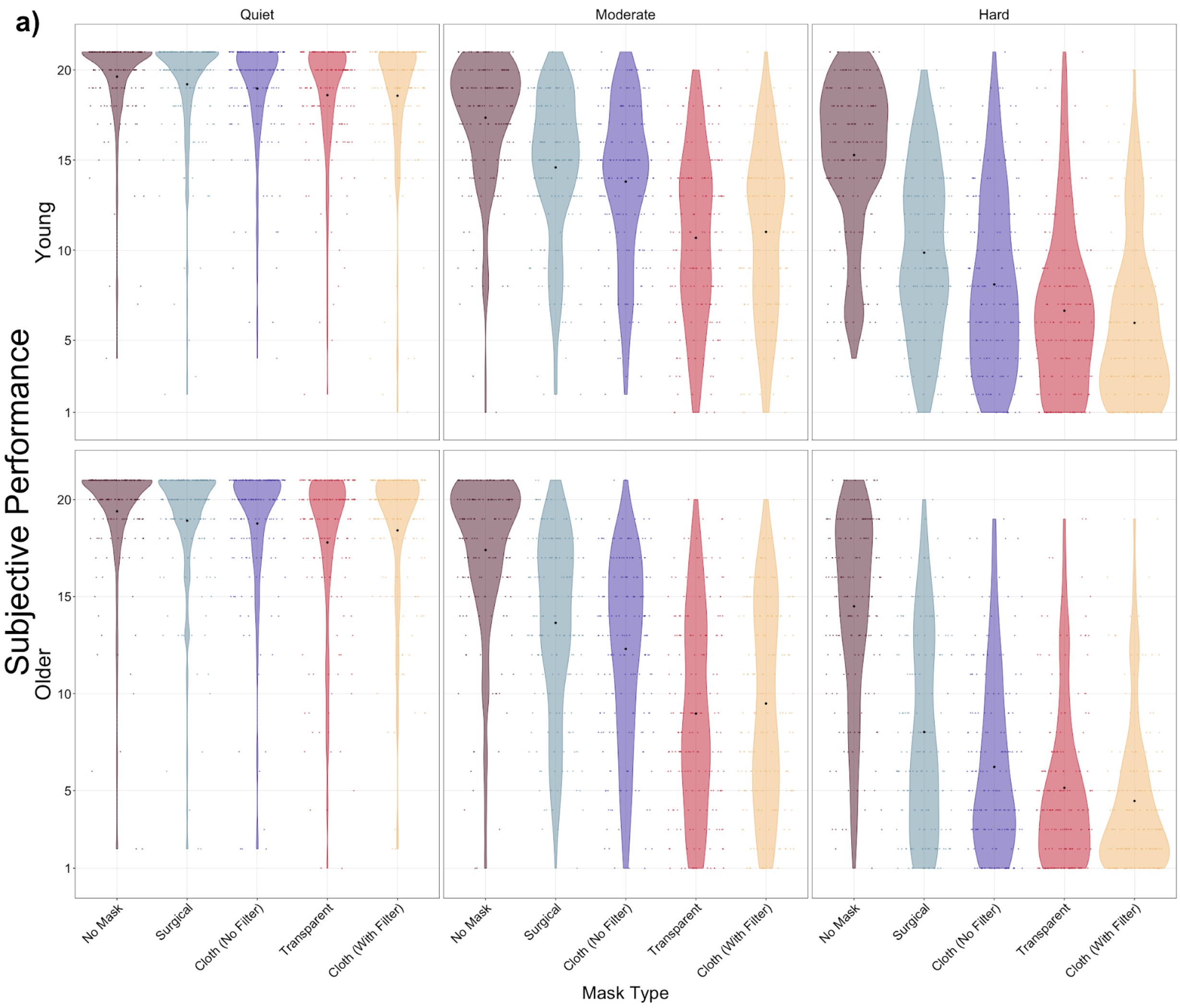
Quiet
Moderate
Hard
Subjective Performance
Young
Older
20
15
10
5
1
No Mask
Surgical
Cloth (No Filter)
Transparent
Cloth (With Filter)
Mask Type

b)

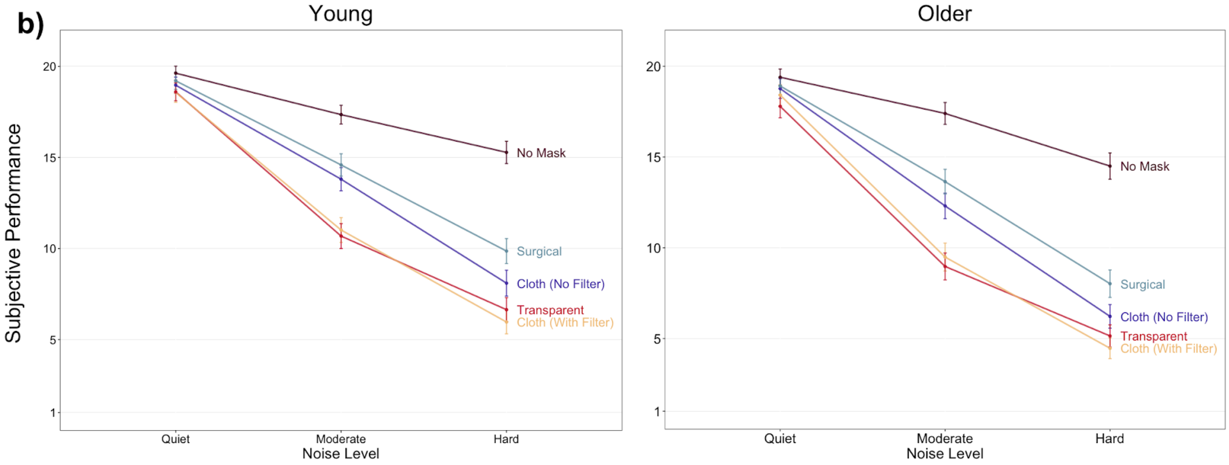
Young
Older
Subjective Performance
20
15
10
5
1
Quiet
Moderate
Hard
Noise Level
No Mask
Surgical
Cloth (No Filter)
Transparent
Cloth (With Filter)

Young Adults

Model comparisons indicated that both fixed effects of interest substantially improved model fit (**Table S1**). Subjective ratings of performance showed a similar pattern of results to the intelligibility data: performance ratings were lower in the moderate relative to quiet condition (B = -5.50, CI = [-5.93, -5.08]) and in the hard relative to the moderate condition (B = -4.32, CI = [-4.72, -3.91]). Further, participants rated their performance highest when the talker was not wearing a mask, and ratings were poorer for the surgical mask and poorer still for the cloth mask without a filter (zero was not contained in the 95% credible interval for any of these pairwise comparisons; see R script for details). Performance ratings were lowest when the talker wore either a transparent mask or a cloth mask with a filter, which did not differ from one another (B = -0.13, CI = [-0.52, 0.27]).

Additional model comparisons indicated that the interaction between mask type and noise level provided a substantial improvement in model fit ($\Delta ELPD$ = -240.6; ΔSE = 23.5). As in the intelligibility analysis, the interaction indicated that the detrimental effects of face masks on subjective performance were exacerbated in more difficult listening conditions (**Figure S1**).

Table S1. *Change in LOO information criterion relative to the best fitting model without an interaction term for the subjective performance analysis in young adults. pid = participant identification number*

	$\Delta ELPD$	ΔSE	Fixed and Random Effects
**Mask and noise model**	0.0	0.0	*mask + noise + (1 + noise|pid)*
**Mask model**	-22.7	9.6	*mask + (1 + noise|pid)*
**Noise model**	-471.3	27.4	*noise + (1 + noise|pid)*

Older Adults

Analyses of the older adult data mirrored those described above using the young adult data. Model comparisons indicated that both mask type and noise level substantially improved model fit (**Table S2**). The subjective data again followed a similar trajectory to the intelligibility data: participants reported reported having poorer performance in the moderate noise level than in quiet (B = -6.29, CI = [-6.80, -5.79]) and in the hard than the moderate noise level (B = -4.69, CI = [-5.10, -4.29]). The 95% credible interval did not contain zero for any of the pairwise comparisons of adjacent mask conditions see R script for details) with the exception of the transparent mask and the cloth mask with a filter (B = 0.15, CI = [-0.29, 0.59]). Finally, the interaction between mask type and noise level (**Figure S1**) provided notable improvements in model fit ($\Delta ELPD$ = -242.6, ΔSE = 23.6).

Table S2. *Change in LOO information criterion relative to the best fitting model without an interaction term for the subjective performance analysis in older adults. pid = participant identification number*

	$\Delta ELPD$	ΔSE	Fixed and Random Effects
**Mask and noise model**	0.0	0.0	*mask + noise + (1 + mask|pid)*
**Mask model**	-33.4	9.5	*mask + (1 + mask|pid)*
**Noise model**	-496.6	29.2	*noise + (1 + mask|pid)*

Constraints on Generality (Simons et al., 2017)

There are a number of points to keep in mind when interpreting our findings. We chose a range of face masks, but other masks—or other examples of the types we chose (e.g., cloth masks produced by a different company)—might lead to different results. Intelligibility and subjective effort are also both

likely to be influenced by the specific stimuli and task. Further, as with all online testing, we expect greater variability in listening environments, participant engagement, and interference from other tasks than would be found in laboratory settings. Although we would expect a similar pattern of results in an in-lab sample, we would expect less variability and therefore more pronounced differences between groups. Finally, we tested young and older adults with self-reported normal hearing, and we expect that the pattern of results may be different for individuals with hearing loss or cochlear implants.

Additional Tables

Table S3. *Young and older adult mean intelligibility (percent correct) and subjective effort ratings (1–21), collapsed across all conditions*

Noise level	Percent correct
YA intelligibility	78.21%
OA intelligibility	76.41%
YA effort	11.55
OA effort	12.91

Table S4. *Young adult mean percent correct for each mask type collapsed across noise levels.*

Mask type	Percent correct
No mask	93.17%
Surgical	81.76%
Cloth (no filter)	78.26%
Transparent	69.84%
Cloth (with filter)	68.04%

Table S5. *Young adult mean percent correct for each noise level collapsed across mask types.*

Noise level	Percent correct
Quiet	95.81%
Moderate	79.41%
Hard	59.41

Table S6. *Older adult mean percent correct for each mask type collapsed across noise levels.*

Mask type	Percent correct
No mask	93.21%
Surgical	79.70%
Cloth (no filter)	75.96%
Transparent	67.73%
Cloth (with filter)	65.48%

Table S7. *Older adult mean percent correct for each noise level collapsed across mask types.*

Noise level	Percent correct
Quiet	96.18%
Moderate	78.18%
Hard	54.89%

Table S8. *Mean (SD) CESD-R for young and older adults.*

	Young adults	Older adults
Mean CESD-R Score	12.44 (11.66)	7.48 (9.57)
Median CESD-R Score	9.00	4.00

Table S9. *Mean (SD) 15iSSQ score and 15iSSQ speech subscore for young and older adults.*

	Young adults	Older adults
15iSSQ Total Score	8.17 (1.24)	8.58 (1.27)
15iSSQ Speech Subscore	7.94 (1.42)	8.12 (1.66)

References

Chandrasekaran, B., Van Engen, K., Xie, Z., Beevers, C. G., & Maddox, W. T. (2015). Influence of depressive symptoms on speech perception in adverse listening conditions. *Cognition & Emotion*, *29*(5), 900–909.

Eaton, W. W., Smith, C., Ybarra, M., Muntaner, C., & Tien, A. (2004). Center for Epidemiologic Studies Depression Scale: Review and Revision (CESD and CESD-R). *The Use of Psychological Testing for Treatment Planning and Outcomes Assessment: Instruments for Adults., Volume 3, 3rd Ed.*, *3*(2004), 363–377.

Gatehouse, S., & Noble, W. (2004). The Speech, Spatial and Qualities of Hearing Scale (SSQ). *International Journal of Audiology*, *43*(2), 85–99.

Koeritzer, M. A., Rogers, C. S., Van Engen, K. J., & Peelle, J. E. (2018). The Impact of Age, Background Noise, Semantic Ambiguity, and Hearing Loss on Recognition Memory for Spoken Sentences. *Journal of Speech, Language, and Hearing Research: JSLHR*, *61*(3), 740–751.

Lee, Y. S., Wingfield, A., Min, N. E., Kotloff, E., Grossman, M., & Peelle, J. E. (2018). Differences in hearing acuity among "normal-hearing" young adults modulate the neural basis for speech comprehension. *eNeuro*, *5*(3), e0263–17.2018.

McCoy, S. L., Tun, P. A., Cox, L. C., Colangelo, M., Stewart, R., & Wingfield, A. (2005). Hearing loss and perceptual effort: Downstream effects on older adults' memory for speech. *The Quarterly Journal of Experimental Psychology*, *58*(1), 22–33.

Moulin, A., Vergne, J., Gallego, S., & Micheyl, C. (2019). A New Speech, Spatial, and Qualities of Hearing Scale Short-Form: Factor, Cluster, and Comparative Analyses. *Ear and Hearing*, *40*(4), 938–950.

Radloff, L. S. (1977). The CES-D Scale: A Self-Report Depression Scale for Research in the General Population. *Applied Psychological Measurement*, *1*(3), 385–401.

Simons, D. J., Shoda, Y., & Lindsay, D. S. (2017). Constraints on Generality (COG): A Proposed Addition to All Empirical Papers. *Perspectives on Psychological Science: A Journal of the*

Association for Psychological Science, *12*(6), 1123–1128.

Tun, P. A., McCoy, S., & Wingfield, A. (2009). Aging, hearing acuity, and the attentional costs of effortful listening. *Psychology and Aging*, *24*, 761–766.
